# Editorials

**Published:** 1903-02

**Authors:** 


### EDITORIALS.

### WHAT IS THE MATTER WITH THE VETERINARIANS OF NEW YORK STATE?

On the 11th of February the New York State Senate and Assembly Agricultural Committee held a joint meeting for the purpose of hearing arguments for and against a bill to require a clean bill of health covering neat cattle shipped into the State for dairy or breeding purposes.

This is a bill that is of vital importance to the health of the live-stock of New York State. A number of States have laws of this description. In fact, practically all of the States touching upon New York State to the east and south have such a law, and it has been stated frequently by distinguished veterinarians of New York that the absence of this protection has the effect of bringing to New York State more than her natural proportion of tubercular cows. A great effort is being made all over the world to find a means of repressing tuberculosis of cattle and to put into operation measures that are planned to have this effect. This work is distinctly veterinary work, and the veterinarians are the natural experts on the questions pertaining to it. When the law-making body of a State has before it a project relating to this subject the natural experts to give such a committee the advice and counsel that it must have, if it is to enact wise laws, are veterinarians. But in New York State, for some reasons, the veterinarians are content to occupy a subordinate and humiliating position; a position that should be most humiliating to them and is deplored by the profession in other parts of the country. When this question of inspecting dairy cows and cattle for breeding purposes for the control of tuberculosis was before the Legislature it appears that the chief arguments were made by Senators and Assemblymen, by men representing the Buffalo Live-stock Association, by cow dealers, by the Commissioner of Agriculture, and by the Assistant Commissioner of Agriculture. In the accounts of the hearing that have reached us there is no intimation that the voice of a veterinarian was heard on this distinctly veterinary question.

The first bill signed by the Governor of New York State during the present session of the Legislature was one appropriating $10,000 to the Commission of Agriculture for use in enforcing the quarantine against the New England States on account of foot-and-mouth disease. Why was this money not appropriated to a State Live-stock Sanitary Board, State Veterinary Board, or to a State Veterinarian? For the reason that no such organization or official exists in New York State. Why do they not exist? To answer this question we must first have an answer to the following: What is the matter with the veterinarians of New York State?

### VITALITY AND IMMUNITY.

THE most excellent article now current in the JOURNAL, entitled "Vitality and Immunity," should be read by every JOURNAL subscriber. It is a *résumé* of the most advanced work done at home and abroad along those lines that have shed so much light upon the dark paths of pathology. It is written in a most attractive and readable manner, and is just one of those presentations of a broad subject that should be familiar to every progressive student of comparative medicine. It is destined, when carefully considered, to awaken a broader, deeper, and better interest along true lines of preventive medicine than any article we have presented our readers for a long time. Dr. Schulin deserves well at our hands for this timely article, and we trust the JOURNAL readers may be favored again by the wealth of his pen.

